# Supplementary material for: The impact of augmented feedback (and technology) on learning and teaching cricket skill: A systematic review with meta-analysis
Source: PLoS One. 2022 Dec 16;17(12):e0279121. doi: 10.1371/journal.pone.0279121 (PMC9757599; doi:10.1371/journal.pone.0279121)
Supplement: S1 Table — (DOCX) [file pone.0279121.s002.docx]

**S2 Table.** Database search string

| 1 (Activity Specific) |  | 2 (Feedback related) |  | 3 (Exclude) |
| --- | --- | --- | --- | --- |
| cricket* | AND | feedback OR feed-back OR “feed back” OR augment* OR terminal* OR instruct* OR learn* OR practic* OR result* OR perform* OR atten* OR observ* OR concurrent* OR fading OR faded OR visual* OR video* OR verbal* OR summary OR action-effect OR modality OR haptic OR auditory OR display* OR effort* OR intervention OR frequen* OR self-other OR error* OR “intrinsic* motivat*” OR self-modelling OR “constrained action hypothesis” OR explor* OR transitional* | NOT | animal* OR insect* OR gryll* OR militar* OR weapon* OR laser* OR colony OR reproduction OR biology OR chemical OR market* |

Search consisted of a Title or Abstract search for key terms (e.g., TI (cricket*) OR AB(cricket*). Limiters (where available) consisted of Language (English), Publication Type (Journal Article or Article or Academic Journal or Early Access) and Peer Reviewed.
