# Supplementary material for: The impact of augmented feedback (and technology) on learning and teaching cricket skill: A systematic review with meta-analysis
Source: PLoS One. 2022 Dec 16;17(12):e0279121. doi: 10.1371/journal.pone.0279121 (PMC9757599; doi:10.1371/journal.pone.0279121)
Supplement: Attachment — Submitted filename: Response to Reviewers.docx [file pone.0279121.s003.docx]

Dear Editors,

RE: PONE-D-22-15875 entitled “The impact of augmented feedback (and technology) on learning and teaching cricket skill: A systematic review with meta-analysis”

We would like to acknowledge and thank the reviewers for their valuable insights and suggestions about this manuscript. Please see the table below for specific responses to each of the comments.

Kind regards,

The Authors.

**Response to Reviewer Comments**

|  | **Comments** | **Response** | **Action** |
| --- | --- | --- | --- |
| **Editor Comments** | | | |
| **1** | Please ensure that your manuscript meets PLOS ONE's style requirements, including those for file naming | The authors have checked the document and submission to ensure it meets the submission guidelines |  |
| **2** | We note that the grant information you provided in the ‘Funding Information’ and ‘Financial Disclosure’ sections do not match.  When you resubmit, please ensure that you provide the correct grant numbers for the awards you received for your study in the ‘Funding Information’ section | Thank you for the comment. No grant was provided for this submission – the funding information referred to relates to a PhD stipend scholarship funded by the Australian Government that we are acknowledging. |  |
| **3** | Please state what role the funders took in the study.  If the funders had no role, please state: "The funders had no role in study design, data collection and analysis, decision to publish, or preparation of the manuscript."  If this statement is not correct you must amend it as needed.  Please include this amended Role of Funder statement in your cover letter; we will change the online submission form on your behalf | The suggested comment will be included during the submission progress and cover letter to highlight that the funders played no role in the manuscript, given the nature of the funding (i.e., PhD stipend from the Australian Government) | Comment to be added:  The funders had no role in study design, data collection and analysis, decision to publish, or preparation of the manuscript. |
| **4** | In your Data Availability statement, you have not specified where the minimal data set underlying the results described in your manuscript can be found. PLOS defines a study's minimal data set as the underlying data used to reach the conclusions drawn in the manuscript and any additional data required to replicate the reported study findings in their entirety. All PLOS journals require that the minimal data set be made fully available. For more information about our data policy, please see [http://journals.plos.org/plosone/s/data-availability](https://protect-au.mimecast.com/s/v6i8CjZr7Zs0ERD7tn7CgY?domain=journals.plos.org).  Upon re-submitting your revised manuscript, please upload your study’s minimal underlying data set as either Supporting Information files or to a stable, public repository and include the relevant URLs, DOIs, or accession numbers within your revised cover letter. For a list of acceptable repositories, please see [http://journals.plos.org/plosone/s/data-availability#loc-recommended-repositories](https://protect-au.mimecast.com/s/XyWTCk8v98svl5AMtknFj5?domain=journals.plos.org). Any potentially identifying patient information must be fully anonymized.  We will update your Data Availability statement to reflect the information you provide in your cover letter | All data used for the meta-analysis is provided in the manuscript. No additional data is available. |  |
| **5** | We note that this manuscript is a systematic review or meta-analysis; our author guidelines therefore require that you use PRISMA guidance to help improve reporting quality of this type of study. Please upload copies of the completed PRISMA checklist as Supporting Information with a file name “PRISMA checklist” | Thank you for the comment. A completed PRIMSA checklist has been included in the submission (Supplementary Table 1). The file name will be renamed as suggested |  |
| **Reviewer 1** | | | |
| **1** | I find PubMed missing from the databases listed. Of course, they are all selected correctly, and perhaps this would be duplicated, but it is missing for me. | Thank you for the comment. PubMed was not selected as the authors had access to MEDLINE, which encapsulates the largest component of PubMed resources. Therefore, it is anticipated that articles that would have been located through PubMed would have been captured through the MEDLINE database search, in addition to other databases explored. We agree that it would be a duplication. | None required |
| **2** | The section on conclusions is interestingly described. The authors pointed out the fact that the analysis performed was limited. I would suggest creating a separate section on conclusions and a separate section on limitations of the analysis. | The review explores the limitations of the analysis in more detail (Lines 472-476), whilst briefly referring to it in the conclusion section (Lines 498-500) | None required |
| **3** | I suggest accepting the paper for publication with minor corrections | Thank you for the positive comments on our manuscript. | None required |
| **Reviewer 2** | | | |
| **1** | There are a number of limitations which should be included in the manuscript, likely stemming from the lack of feedback specific studies in this area, and include the inability to review the frequency, timing and provision of feedback approaches on cricket skill. The review itself is rather an overview of feedback-involved interventions on skill-based performance outcomes in cricket-specific “research”. | Thank you for the comment. The authors agree and have added to the manuscript to highlight these points relating to the nature of the review (feedback involved interventions) and limitations (feedback attributes) | Nature of review  – Lines 268-270  “Rather, the review focuses on feedback-involved interventions, and their impact on skill-based performance outcomes within cricket-specific research.”  – Lines 477-479  “Rather, feedback-involved interventions and subsequent impact on skill-based performance outcomes within cricket-specific research was explored.”  Limitations of feedback attributes  – Lines 479-481  “Such limitations in exploring key attributes of feedback (such as the timing, frequency and type) may have further impacted homogeneity of the studies and subsequent findings.” |
| **2** | It was disappointing to see that all studies in this space have only involved male cricketers. It is suggested that this point be highlighted, and include a call for future research in this space to include both male and female cricketers. | This point has been added as a recommendation to guide future research | Future directions recommendation  – Lines 487-489  “Further, the review highlighted the limited cricket cohort explored; identifying that future research should focus on both male and female cricketers to best understand the value of feedback practices.” |
| **3** | Specific Comments Line 33-36: Suggest rephrase (For example, smart device applications that enable quick analysis of movement patterns, such as the bat lift in cricket [3], providing augmented information that can be delivered as feedback (either directly to a player or by a coach), influencing how practice can translate into improved performance outcomes during competition) | Thank you for the comment. The sentence has been amended to have better flow | Rephrase  – Lines 33-37  “For example, smart device applications that enable quick analysis of movement patterns, such as the bat lift in cricket [3], provide augmented information that can be delivered as feedback (either directly to a player or by a coach). This extrinsic information can subsequently influence how practice can translate into improved performance outcomes during competition [4].” |
| **4** | Line 39: Please include: It “can” be delivered… after an action, (that is, it can also be provided during an action; also referenced in line 42 and again line 110 | The sentence has been clarified | Clarification  – Lines 40-41  “It can be delivered to a learner during or after an action is completed, putatively, to improve performance during later actions [4].” |
| **5** | Could the authors explain why the timing of feedback (concurrent or terminal) was not delimited in table 1, unlike how the feedback type was included? | The timing of feedback is somewhat explored under the heading Feedback provided (i.e., it alludes to following a trial or video replay). The explicit nature of when feedback was provided was not explored given the limited scope of the studies included in the review (i.e., not standalone feedback studies which did not explore the timing as a key attribute of the feedback delivered). | None required |
| **6** | Could the lack of significance within the results be due to the fact that few included studies were designed to examine the effect of feedback on cricket performance? | The authors agree that the potential lack of significance may be due to the limited nature of how feedback was implemented and explored in the included research studies and would be something to investigate in further research.  This limitation is included in the discussion section (Lines 318-320 and Lines 342-343) | None required |
| **7** | Line 317 – The authors state “In considering why there was a lack of significance, another possibility may be that the feedback provided was not sufficiently guiding in drawing attention to key information sources.” Could this not also be due to only a few papers specifically examining the influence of feedback on performance? | The author’s agree this could be the case. Please refer to our response to reviewer 2, point 6 above | None required |
| **8** | Line 475 “Additionally, of the included studies, none explicitly explored the role of isolated augmented feedback.” Strongly suggested to include in limitations that the timing, frequency and method of providing feedback was not assessed in this study which may also have impacted homogeneity of the studies and subsequent findings. | Thank you for the suggestion. The suggested comment has been included to identify these limitations within the discussion. | Comment on limitation  – Lines 479-481  “Such limitations in exploring key attributes of feedback (such as the timing, frequency and type) may have further impacted homogeneity of the studies and subsequent findings.” |
| **9** | Line 489: “the role of augmented feedback within the sport of cricket.” This review is just focused on research conducted within the sport of cricket | The sentence has been amended to reflect this point. | Clarification  – Lines 494-495  “To the authors knowledge, this is the first systematic review or meta-analysis that has explored the role of augmented feedback within research for the sport of cricket.” |
| **10** | It is recommended the manuscript be reviewed generally to improve the quality of some sentence structures. (e.g., line 464: Given the continual advances to technology, including within sport settings, exciting opportunities exist for the development and use of technology which can be embedded within representative practice environments and, that supports coaches to present augmented feedback in-situ which helps guide athlete exploration processes) | Thank you for the comment – the manuscript has been reviewed to assess sentence flow as advised. The highlighted sentence has also been refined as suggested. | Rephrase  – Lines 465-469  “Given the continual advances to technology, including within sport settings, exciting opportunities exist for the development and use of technology which can be embedded within representative practice environments. Such technologies can support coaches to present augmented feedback in-situ; an important consideration which may help guide athlete exploration processes.” |
